# Proteasome activator PA200 regulates myofibroblast differentiation

**DOI:** 10.1038/s41598-019-51665-0

**Published:** 2019-10-23

**Authors:** Vanessa Welk, Thomas Meul, Christina Lukas, Ilona E. Kammerl, Shrikant R. Mulay, Andrea C. Schamberger, Nora Semren, Isis E. Fernandez, Hans-Joachim Anders, Andreas Günther, Jürgen Behr, Oliver Eickelberg, Martina Korfei, Silke Meiners

**Affiliations:** 1 Comprehensive Pneumology Center (CPC), University Hospital of the Ludwig-Maximilians-University (LMU) and Helmholtz Zentrum München, Member of the German Center for Lung Research (DZL), Max-Lebsche Platz 31, 81377 Munich, Germany; 2 Division of Nephrology, Medizinische Klinik und Poliklinik IV, Klinikum der Universität, Ziemssenstraße 1, 80336 Munich, Germany; 3 Translational Lung Research and CPC-M bioArchive, Helmholtz Zentrum München, Comprehensive Pneumology Center Munich DZL/CPC-M, Munich, Germany; 4 Department of Internal Medicine, Universities of Giessen and Marburg Lung Center (UGMLC), Justus-Liebig-University Giessen, Member of the German Center for Lung Research (DZL), Giessen, Germany; 5 European IPF Network and European IPF Registry, Giessen, Germany; 6 Asklepios Fachkliniken München-Gauting, Gauting, Germany; 7 Medizinische Klinik und Poliklinik V, Klinikum der Ludwig-Maximilians-Universität, Member of the DZL, Munich, Germany; 8 Division of Pulmonary Sciences and Critical Care Medicine, University of Colorado, 12605 E. 16th Ave, Aurora, CO 80045 United States

**Keywords:** Respiratory tract diseases, Molecular medicine

## Abstract

The proteasome is essential for the selective degradation of most cellular proteins and is fine-tuned according to cellular needs. Proteasome activators serve as building blocks to adjust protein turnover in cell growth and differentiation. Understanding the cellular function of proteasome activation in more detail offers a new strategy for therapeutic targeting of proteasomal protein breakdown in disease. The role of the proteasome activator PA200 in cell function and its regulation in disease is unknown. In this study, we investigated the function of PA200 in myofibroblast differentiation and fibrotic tissue remodeling. PA200 was upregulated in hyperplastic basal cells and myofibroblasts of fibrotic lungs from patients with idiopathic pulmonary fibrosis. Increased expression of PA200 and enhanced formation of PA200-proteasome complexes was also evident in experimental fibrosis of the lung and kidney *in vivo* and in activated primary human myofibroblasts of the lung *in vitro*. Transient silencing and overexpression revealed that PA200 functions as a negative regulator of myofibroblast differentiation of human but not mouse cells. Our data thus suggest an unexpected and important role for PA200 in adjusting myofibroblast activation in response to pro-fibrotic stimuli, which fails in idiopathic pulmonary fibrosis.

## Introduction

Idiopathic pulmonary fibrosis (IPF) is a devastating, progressive interstitial lung disease with a median survival of patients of 3-5 years^1^. So far no effective therapies exist. Fibrotic lung remodeling is thought to be initiated by repetitive micro-injuries to the lung epithelium that triggers an aberrant wound healing response, which involves uncontrolled myofibroblast activation and excessive deposition of extracellular matrix (ECM) impairing lung function and causing organ failure^2,3^.

Proteasomes are intracellular protease complexes involved in the degradation of most cellular proteins, including unwanted, misfolded and aged proteins. They function as essential regulators of cellular growth and differentiation^4^. The proteolytic activity resides in the barrel-shaped 20S proteasome that associates with different activators to stimulate entry of proteins and peptides for degradation^5–7^. Four proteasome activators have been identified, the 19S regulator and the alternative proteasome activators PA28αβ, PA28γ, and PA200, that bind the 20S proteasome either at one or both of its symmetric ends^7^. Attachment of one or two 19S regulators results in the formation of 26S proteasomes responsible for ubiquitin- and ATP-dependent protein breakdown^8^. Alternative proteasome activators function in an ATP- and ubiquitin-independent manner, their exact function, however, is not fully understood yet^7^. Proteasome activators are proposed to function as building blocks that are recruited to the 20S core particle to allow adaptive regulation of proteasome function according to cellular needs^7,9–11^.

The proteasome activator PA200 is a 200 kDa, monomeric protein expressed in both the cytosolic and nuclear compartment. It is composed of HEAT repeats forming a dome-like cap that binds to the 20S core particle but also attaches to 26S proteasome complexes forming so-called hybrid proteasomes^12–14^. PA200 has been shown to be involved in DNA repair and degradation of acetylated histones^15–17^. Its exact molecular function as well as its regulation in disease or upon certain cellular stimuli, however, is still elusive.

We recently observed that activation and differentiation of pro-fibrotic myofibroblasts in fibrotic tissue remodeling of the lung requires the increased assembly and activity of 26S proteasomes^18^. Regulation of alternative proteasome complexes has not been investigated in this context so far. The present study focuses on the role of PA200 in myofibroblast differentiation and fibrotic tissue remodeling.

## Results

### PA200 is upregulated in idiopathic pulmonary fibrosis

**Figure 1:**
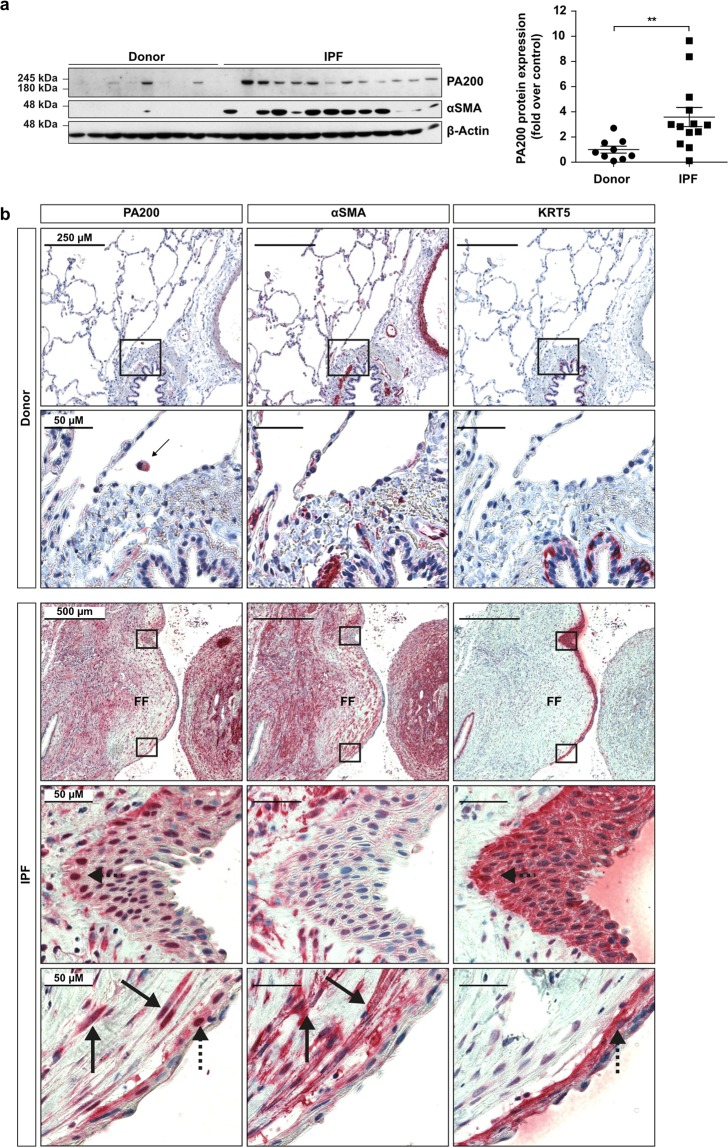
PA200 is upregulated in IPF lungs. (**a**) Homogenates of human IPF and donor lung tissues were analyzed for PA200 and α-smooth muscle actin (αSMA) expression by Western blotting. Diagram shows densitometric analysis of PA200 expression relative to β-Actin levels and normalized to the mean signal obtained for donor tissues (Mann-Whitney U test, donor tissues *n* = 9, IPF tissues *n* = 13). (**b**) Representative immunohistochemistry staining of PA200, αSMA and cytokeratin-5 (KRT5) in serial lung sections from donors (*n* = 6) and IPF patients (*n* = 10). Arrows indicate PA200 overexpression in αSMA expressing myofibroblasts of fibroblast foci (FF) in IPF lungs. Dashed arrows indicate PA200 overexpression in KRT5 expressing bronchiolar basal cells in IPF lungs. Full-length immunoblots are presented in Supplementary Fig. S5.

PA200 protein levels were analyzed in explanted lung tissues from IPF patients and controls by Western blotting and immunohistochemistry. We first validated the specificity of the most commonly used anti-PA200 antibody targeted against amino acids 1620-1634 of the human protein. Using PA200-silenced human lung cells and testis tissue of PA200^−/−^ mice, we observed that this antibody unspecifically recognizes a protein species of 160 kDa, which has previously been proposed as a PA200 isoform^12^ (Supplementary Fig. S1A–C). Further screening of antibodies identified other commercially available anti-PA200 antibodies that specifically detected PA200 by Western blotting or immunohistochemistry (Supplementary Fig. S1D–F). When we applied these antibodies for Western blot analysis of human lung tissues, we observed that expression of PA200 was significantly upregulated in lungs of IPF patients compared to donors (Fig. 1A). Immunohistochemistry analysis identified increased expression of PA200 in well-known IPF driver cells, i.e. α smooth muscle actin (αSMA)-positive myofibroblasts^3^ and keratin 5 (KRT5)-positive hyperplastic bronchial basal cells^19^ (Fig. 1B and Supplementary Fig. S2). In contrast, PA200 was expressed only at low basal levels in normal donor lungs.

### Increased formation of PA200-proteasome complexes in experimental fibrosis of lung and kidney

**Figure 2:**
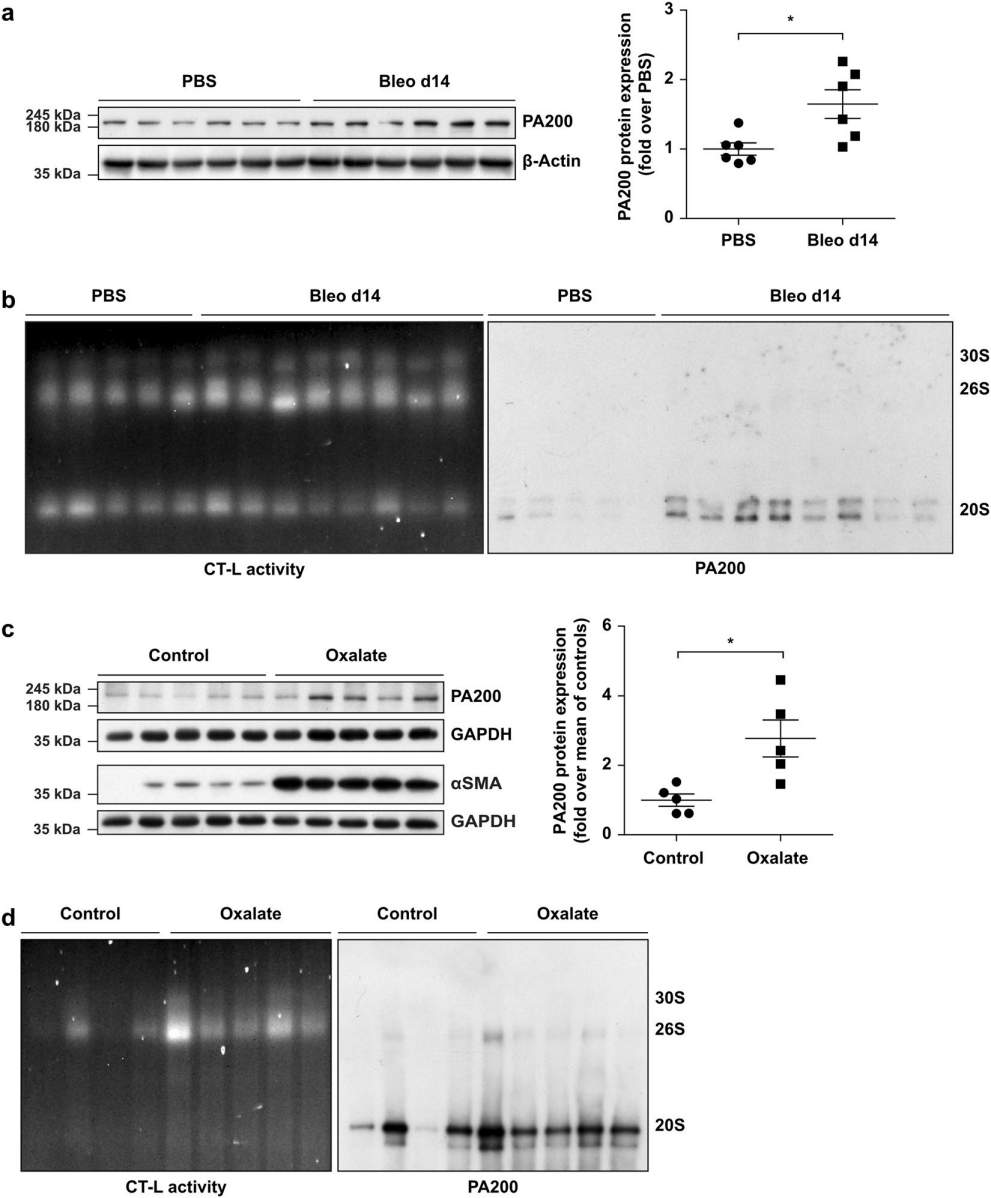
Augmented formation of alternative PA200-proteasome complexes in experimental fibrosis. (**a**) PA200 protein expression in lung homogenates of bleomycin- or PBS-instilled mice with densitometric analysis of PA200 signals normalized to mean of PBS-treated controls. β-Actin served for normalization of the signal (Mann-Whitney U test, *n* = 6 per group). (**b**) Native gel analysis of native lung homogenates of bleomycin- and PBS-instilled mice with substrate overlay for the chymotrypsin-like (CT-L) proteasome activity and immunoblotting for PA200. (**c**) PA200 and αSMA protein expression in kidney RIPA homogenates of oxalate-fed mice and controls with densitometric analysis of PA200 normalized to mean of controls. GAPDH served for normalization of the signal (Mann-Whitney U test, *n* = 5 per group). (**d**) Native gel analysis of kidney extracts from oxalate-treated and control mice with substrate overlay for CT-L activity and immunoblotting for PA200. Full-length immunoblots are presented in Supplementary Fig. S5.

We investigated the formation of alternative PA200-proteasome complexes in fibrotic remodeling of the lung using bleomycin-induced lung fibrosis in mice^18,20^. Similar to IPF tissue, we observed significant upregulation of PA200 protein levels in fibrotically remodeled lungs 14 days after bleomycin instillation (Fig. 2A). Furthermore, native gel analysis and subsequent immunoblotting revealed that PA200 was effectively recruited to the 20S proteasome to form alternative PA200-proteasome complexes (Fig. 2B). We also noted pronounced induction of PA200 expression as well as formation of PA200-proteasome complexes in an experimental model of kidney fibrosis (Fig. 2C,D). Fibrotic remodeling of the kidney was provoked by feeding mice with an oxalate-rich diet and confirmed by Masson-Trichrome staining for collagens (Supplementary Fig. S3A) and elevated protein levels of αSMA^21^ (Fig. 2C). Fibrotic kidney remodeling also involved the marked induction of 26S proteasome activity as shown previously for fibrotic remodeling of the lung^18^ (Fig. 2D and Supplementary Fig. S3B). Taken together, these data suggest that increased formation of alternative PA200-proteasome complexes is an inherent feature of fibrotic tissue remodeling.

### PA200 does not protect from bleomycin-induced lung fibrosis in mice

**Figure 3:**
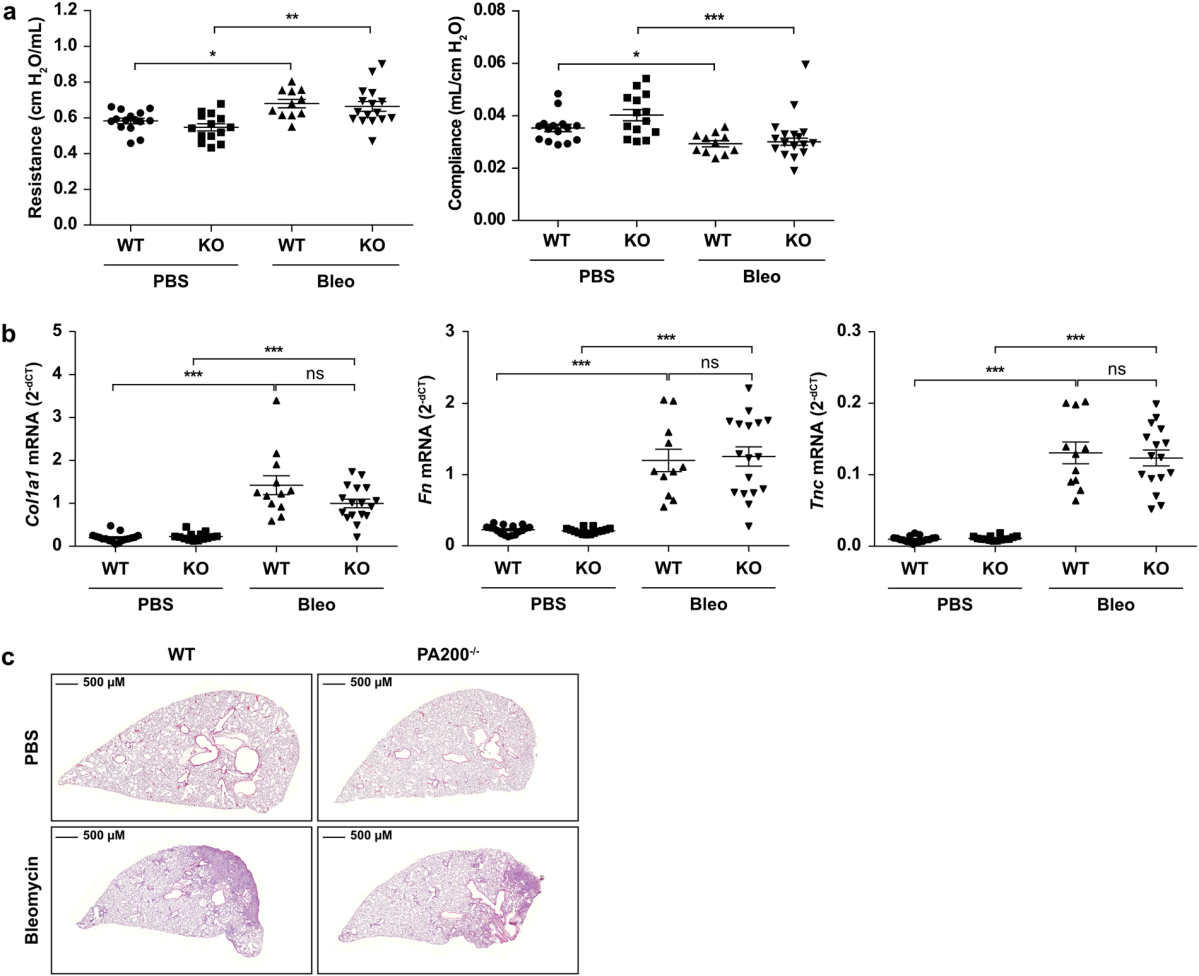
PA200 does not protect from bleomycin-induced lung fibrosis in mice. Lungs of PA200−/− and wildtype (WT) mice instilled with 2 U/kg body weight bleomycin (Bleo) or PBS as control were analyzed for the development of fibrosis at day 14. (**a**) Lung function analysis including resistance and compliance of wildtype (WT) and PA200−/− (KO) mice at day 14 after bleomycin (Bleo) or PBS instillation (Kruskal-Wallis test and Dunn’s multiple comparisons test, *n* = 11–16 per group). (**b**) Lungs of wildtype (WT) and PA200−/− (KO) mice 14 days after PBS- or bleomycin-instillation were analyzed for fibrotic marker collagen1α1 (*Col1a1*), fibronectin (*Fn*) and tenascin C (*Tnc*) mRNA expression by RTqPCR. *Rpl19* served as housekeeping gene (Kruskal-Wallis test and Dunn’s multiple comparisons test, *n* = 12–17 per group; combined results from two independent animal experiments). (**c**) Representative hematoxylin & eosin staining of paraffin-embedded lung tissue sections at day 14 after bleomycin instillation.

To assess the causal contribution of PA200 to fibrotic tissue remodeling, we induced lung fibrosis in PA200^−/−^ mice by a single instillation of bleomycin into the lungs and analyzed lung function and histology in comparison to wild type littermates. Development of lung fibrosis was not grossly altered in PA200^−/−^ mice as assessed by lung function parameters such as resistance and compliance, profibrotic marker gene expression, and hematoxylin & eosin staining (Fig. 3A–C). These data indicate that deficiency of PA200 does not have a major impact on bleomycin-induced lung fibrosis in mice.

### Induction of PA200-proteasome complexes in myofibroblast differentiation

**Figure 4:**
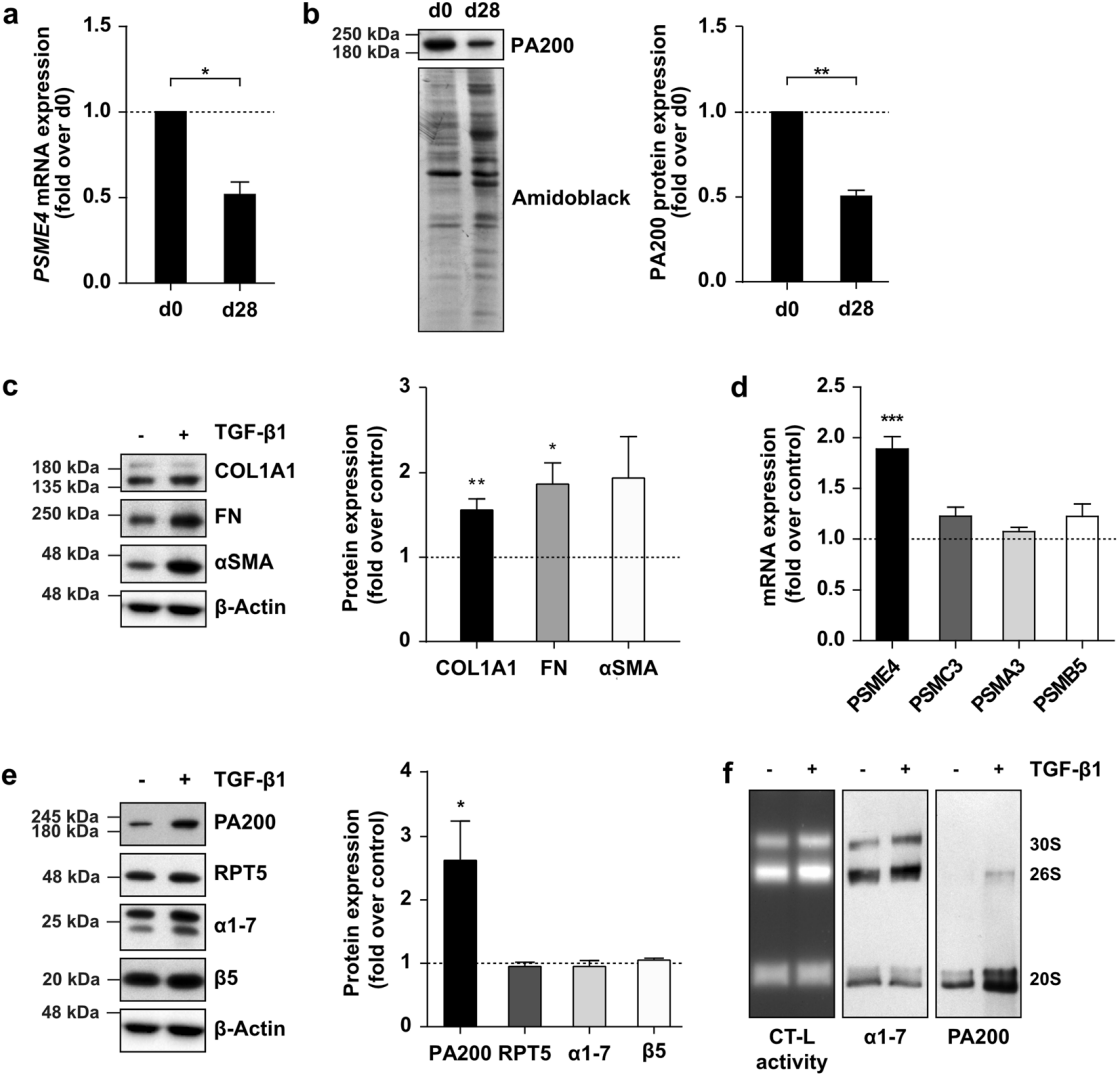
Increased expression of PA200 in primary human bronchial basal cells and TGF-β1 activated myofibroblasts. (**A**) PA200 *(PSME4)* mRNA levels analyzed in primary human bronchial basal cells at day 0 (d0) and day 28 (d28) of differentiation into bronchial epithelial cells. *RPL19* served as housekeeping gene (one-sample t-test, primary human bronchial basal cells from *n* = 3 different patients). (**b**) Analysis of PA200 protein expression in cells analyzed in (**a**). Amidoblack staining served as a loading control for Western blotting (one-sample t-test, primary human bronchial basal cells from *n* = 3 different patients). (**c**) Primary human lung fibroblasts (phLF) treated with TGF-β1 for 48 h analyzed for myofibroblast markers collagen1α1 (COL1A1), fibronectin (FN) and α-smooth muscle actin (αSMA) protein expression with densitometric analysis of protein levels relative to housekeeping protein β-Actin normalized to controls (one-sample t-test, in phLF from *n* = 6 organ donors). (**d**) Cells analyzed in (**c**) were assessed for PA200 (*PSME4*), 19S regulator subunit RPT5 (*PSMC3*) and 20S proteasome subunits α7 (*PSMA3*) and β5 (*PSMB5*) mRNA expression. *RPL19* served as housekeeping gene (one-sample t-test, in phLF from *n* = 6 organ donors). (**e**) Protein expression of PA200, RPT5, α1-7 and β5 in cell extracts analyzed in (**c**) with densitometric analysis of protein levels relative to housekeeping protein β-Actin normalized to controls (one-sample t-test, in phLF from *n* = 6 organ donors). (**f**) Native gel analysis of native extracts of TGF-β1-treated phLF with substrate overlay for the chymotrypsin-like (CT-L) proteasome activity and immunoblotting for PA200. Figure shows representative results of experiments performed in phLF from *n* = 3 organ donors. Full-length immunoblots are presented in Supplementary Fig. S5.

Despite these negative *in vivo* data, we continued to analyze the function of PA200 in primary human cells of the lung, focusing on the two main profibrotic cell types with elevated PA200 expression in IPF tissue, i.e. bronchial basal cells and myofibroblasts. We first analyzed PA200 expression in primary bronchial basal cells isolated from lung organ donors and compared it to primary bronchial epithelial cells after 28 days of *in vitro* differentiation at air-liquid interface^22^. Expression of PA200 was almost twofold higher on transcript and protein level in undifferentiated basal cells compared to fully differentiated bronchial epithelial cells (Fig. 4A,B), which corresponded well to the elevated levels of PA200 in hyperplastic basal cells in IPF. Next, we investigated regulation of PA200 in primary human lung fibroblasts (phLF) in response to transforming growth factor (TGF)-β1 as the major profibrotic cytokine^23^. Treatment of phLF with TGF-β1 for 48 h increased expression of the myofibroblast marker αSMA and of the extracellular matrix proteins fibronectin (FN) and collagen1α1 (COL1A1) (Fig. 4C). Of note, TGF-β1 specifically upregulated expression of PA200 on the transcript and protein level (Fig. 4D,E). In contrast, other subunits of the 26S proteasome, such as the 19S regulator subunit RPT5 (gene name: PSMC3), the 20S subunits α1-7 and the 20S catalytic subunit β5 (gene name: PSMB5), were not regulated (Fig. 4D,E). Moreover, not only the expression but also the formation of alternative PA200-proteasome complexes was augmented upon myofibroblast differentiation (Fig. 4F). PA200 was found to be mainly associated with 20S proteasome complexes and to a lesser extent with the 26S proteasome forming hybrid complexes. These data demonstrate that PA200 is upregulated by the profibrotic stimulus TGF-β1 in the process of myofibroblast differentiation.

### PA200 is a negative regulator of myofibroblast activation

**Figure 5:**
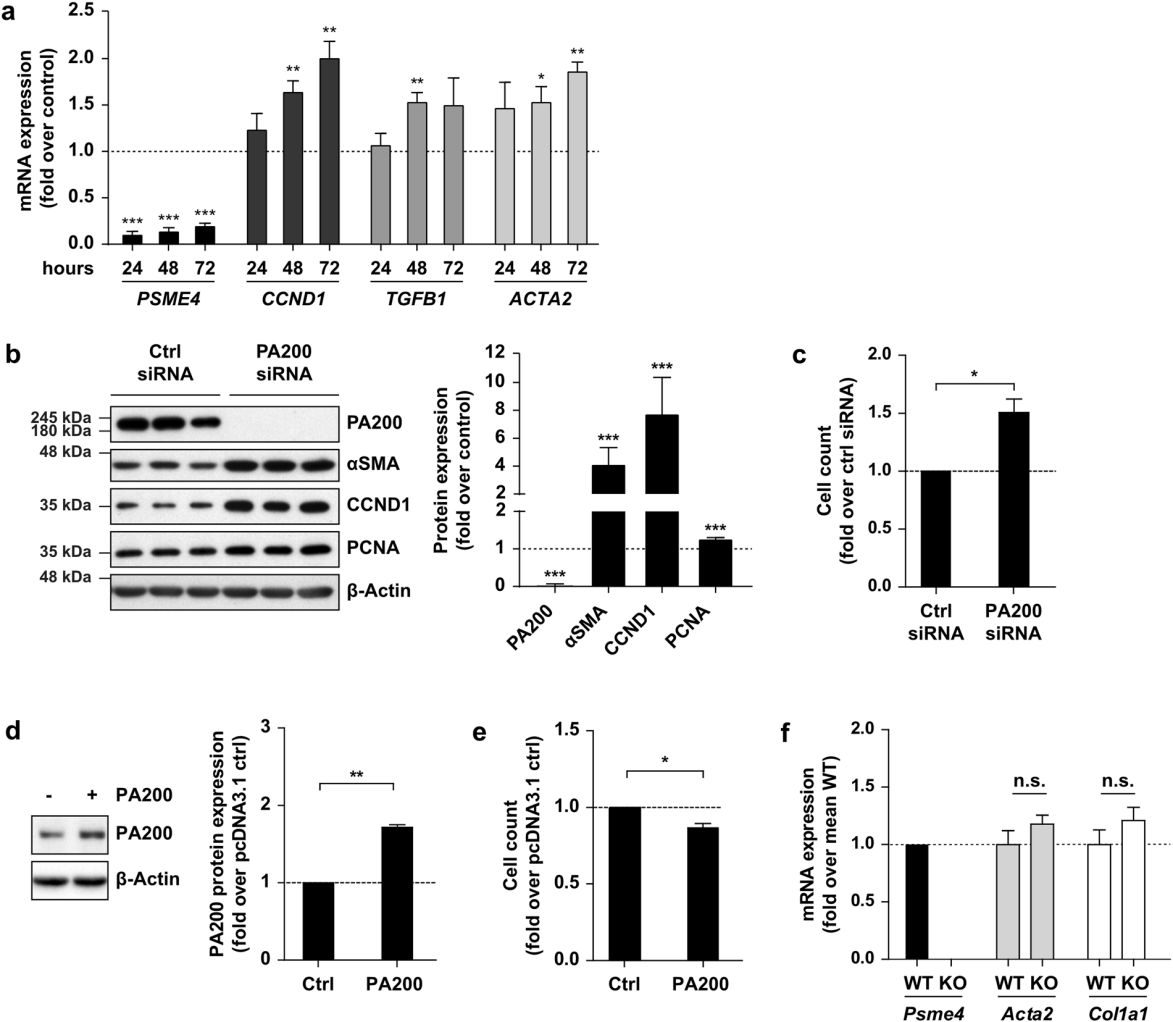
PA200 is a negative regulator of myofibroblast differentiation. (**a**) mRNA expression analysis of PA200 (*PSME4*), cyclin D1 (*CCND1*), TGF-β1 (*TGFB1*) and αSMA (*ACTA2*) in phLF after 24, 48 and 72 h of PA200 silencing. *HPRT* served as housekeeping gene and expression was normalized to time-matching controls (one-sample t-test, phLF from *n* = 3 organ donors). (**b**) Protein expression analysis of PA200, myofibroblast marker α-smooth muscle actin (αSMA) and proliferation markers cyclin D1 (CCND1) and proliferating cell nuclear antigen (PCNA) in phLF transfected with PA200 or control siRNAs for 72 h. Bar diagram shows densitometric analysis of Western blots with normalization of obtained signals to control siRNA-transfected phLF of the same donor (one sample t-test, phLF from *n* = 4 different organ donors). (**c**) Cell count of phLF analyzed 72 h after PA200 silencing and normalized to mean of control siRNA-transfected cells (Mann-Whitney U test, phLF from *n* = 4 different organ donors with three technical replicates per condition). (**d**) Analysis of PA200 protein levels in phLF upon PA200 overexpression for 24 h (one-sample t-test, phLF from *n* = 3 different organ donors). (**e**) Analysis of cell count in phLF under the same conditions used in (**d**) (one-sample t-test, phLF from *n* = 3 different organ donors). (**f**) mRNA expression analysis of *Psme4* (PA200), and myofibroblast markers *Acta2* (αSMA) and *Col1a1* in wildtype and PA200−/− primary mouse lung fibroblasts. *Hprt* served as house keeping gene (Mann-Whitney U test, pmLF from *n* = 4 animals per group). Full-length immunoblots are presented in Supplementary Fig. S5.

To dissect the functional role of PA200 in fibroblasts, we silenced PA200 in phLF. To our surprise, PA200 strongly induced myofibroblast differentiation as demonstrated by increased expression of the proliferation marker cyclin D1 and the myofibroblast markers αSMA and TGF-β1 over time (Fig. 5A). Protein levels of αSMA, proliferation markers cyclin D1, and proliferating cell nuclear antigen (PCNA) were also significantly upregulated 72 h after PA200 silencing (Fig. 5B). Accordingly, PA200 depletion resulted in enhanced fibroblast proliferation as shown by increased cell count (Fig. 5C). In contrast, an only 2-fold overexpression of PA200 in phLF for 24 h led to a significant reduction in cell proliferation (Fig. 5D,E). We were puzzled about this strong phenotype of PA200 silencing in human lung fibroblasts in light of the lack of phenotype for PA200^−/−^ mice in bleomycin-induced lung fibrosis and speculated that there might be species-specific differences. To investigate this hypothesis further, we analyzed primary mouse lung fibroblasts (pmLF) isolated from PA200^−/−^ mice for myofibroblast marker gene expression compared to pmLF isolated from wildtype littermates. Of note, deficiency of PA200 in these mouse lung fibroblasts did not result in any change of αSMA and collagen1α1 gene expression (Fig. 5F). In order to investigate if chronic depletion of PA200 leads to a compensatory effect in knockout mice and thereby conceals the function of PA200 as a negative regulator of myofibroblast differentiation we also performed siRNA-mediated PA200 silencing experiments in pmLF isolated from wildtype mice (Supplementary Fig. S6). In contrast to our results obtained with primary human lung fibroblasts, we did not observe any effect of PA200 silencing on profibrotic marker gene expression in murine lung fibroblasts. These data point towards a species-specific effect for PA200 in myodifferentiation of human fibroblasts.

### PA200 regulates myofibroblast activation beyond TGF-β1 signaling

**Figure 6:**
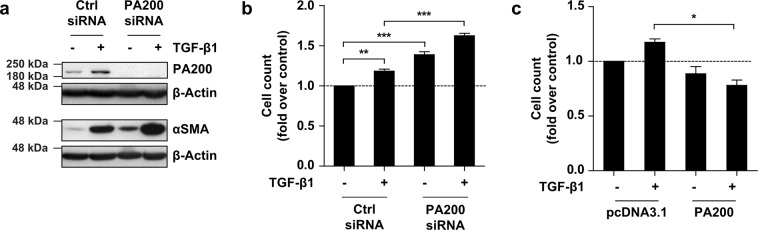
PA200 deficiency augments TGF-β1-induced myofibroblast activation. (**a**) Western blot analysis of PA200 and αSMA of phLF after silencing of PA200 for 24 h and subsequent treatment with TGF-β1 for 48 h. Figure shows representative Western blots of experiments performed with phLF from *n* = 4 donors. (**b**) Analysis of cell count of phLF treated as in (**a**) normalized to control of phLF from the same donor (Bonferroni’s multiple comparison test, phLF from *n* = 3 different organ donors). (**c**) Analysis of cell count of phLF treated 24 h after overexpression of PA200 with TGF-β1 for another 24 h. Cell numbers were normalized to the control of the same donor (Bonferroni’s multiple comparison test, phLF from *n* = 3 different organ donors). Full-length immunoblots are presented in Supplementary Fig. S5.

The induction of PA200 by TGF-β1 on the one hand and its role as a negative regulator of myofibroblast differentiation on the other can be reconciled if assuming that PA200 functions in a negative feedback loop to limit TGF-β1-induced myofibroblast activation. *In silico* analysis of the human PA200 promoter for conserved binding sites of the TGF-β1 responsive SMAD transcription factors, however, did not reveal any evidence for direct activation of the human PA200 promoter by TGF-β1 (data not shown). This is in line with the delayed upregulation of PA200 after 48 h (Fig. 4D,E) and suggests that PA200 is not directly regulated by TGF-β1. To corroborate this finding, we co-treated PA200-silenced phLF with TGF-β1 and assayed myofibroblast activation. Of note, co-treatment of PA200-silenced cells with TGF-β1 further stimulated myofibroblast marker gene expression when compared to PA200 expressing controls (Fig. 6A and Supplementary Fig. S4A). Moreover, cellular proliferation was significantly increased upon TGF-β1 stimulation in PA200-depleted cells when compared to control siRNA-transfected cells (Fig. 6B). In contrast, PA200 overexpression prevented TGF-β1-induced cell proliferation confirming its function as a negative regulator of myofibroblast proliferation (Fig. 6C and Supplementary Fig. S4B). The additive effect of TGF-β1 treatment and PA200 silencing suggests that PA200 regulates myofibroblast activation to some extent independently of the TGF-β1 signaling pathway to limit myofibroblast differentiation.

## Discussion

In the present study, we observed enhanced formation of alternative PA200-proteasome complexes in fibrotic tissue remodeling of lung and kidney and localized increased PA200 expression to hyperplastic basal cells and myofibroblasts in human IPF tissues. In accordance with this observation, PA200 expression was higher in undifferentiated basal cells compared to *in vitro-*differentiated bronchial epithelial cells and PA200-proteasome complexes were upregulated upon TGF-β1-induced myofibroblast differentiation. Unexpectedly, PA200 acted as a negative regulator of myofibroblast differentiation and partly independent of TGF-β1. This effect was specific for primary human cells and not observed in PA200^−/−^ mice. Hence, we propose that in primary human lung fibroblasts PA200 adjusts myofibroblast activation in response to pro-fibrotic stimuli, which fails in idiopathic pulmonary fibrosis.

Our immunohistochemical staining of IPF lungs revealed upregulation of PA200 in driver cells of fibrotic tissue remodeling^3,19^. For this analysis, we used anti-PA200 antibodies that had been validated for their specific detection of PA200 using siRNA-mediated gene silencing in cells and tissue from PA200^−/−^ mice. We would like to stress our validation effort as the most commonly used, commercially available anti-PA200 antibody turned out to unspecifically recognize proteins not related to PA200, which questions some of the published data^12^. PA200 was upregulated in hyperplastic basal cells overlying fibroblast foci. These KRT5-positive basal cells were recently described as a characteristic feature of IPF tissues^19,24^. We observed downregulation of PA200 in the course of basal cell differentiation *in vitro* indicating that elevated levels of PA200 are a specific feature of basal cells compared to differentiated bronchial epithelial cells. Expression of PA200 was also increased in myofibroblasts which drive excessive formation of connective tissue in tissue fibrosis^25,26^. Moreover, PA200 was upregulated by the pro-fibrotic cytokine TGF-β1 in primary human lung fibroblasts resulting in augmented formation of PA200-proteasome complexes in differentiated myofibroblasts. These results indicated that alternative PA200-proteasome complexes may promote myofibroblast activation and fibrotic tissue remodeling. Silencing and overexpression of PA200 in phLF, however, revealed that PA200 negatively regulated myofibroblast differentiation. These data suggest that PA200-proteasome complexes serve to limit myofibroblast differentiation similar to other components of the ubiquitin-proteasome system^27^. We speculate that this negative regulation of myofibroblast activation fails in fibrotic lungs of IPF patients and that PA200 is upregulated in a frustrated attempt to limit myofibroblast activation. The additive effect of PA200 silencing and TGF-β1 treatment on myofibroblast activation indicates that PA200 regulates myofibroblast differentiation partly independent of TGF-β1 signaling pathways. In contrast to the acute silencing of PA200 in primary human fibroblasts, we neither observed increased myofibroblast differentiation in chronically PA200-depleted or acutely PA200-silenced primary mouse lung fibroblasts nor any effect on bleomycin-induced lung fibrosis in PA200^−/−^ mice suggesting that PA200 function is possibly species-specific or somehow compensated in these knockout mice as observed in several other knockout strains^28^. In line with this observation, PA200^−/−^ mice lack any phenotypic defects except for their male infertility^29^. Even when crossed with p53^−/−^ mice, which exhibit increased tumor development, PA200 deficiency did not give rise to any additional phenotype^29^. Moreover, embryonic stem cells isolated from PA200^−/−^ mice did not show any altered sensitivity to bleomycin-induced DNA damage compared to wildtype cells^29^.

PA200 has originally been described as an activator of the caspase-like activity of the proteasome by *in vitro* assays using PA200 purified from bovine testis^12^. A recent publication on recombinant human PA200-proteasome complexes, however, unambigiously shows activation of the trypsin-like activity of the proteasome^30^. PA200^−/−^ mice, however, do not show any obvious alterations in proteasome activity neither in the lung nor in testis (data not shown). Accordingly, PA200 has also been suggested to serve as a proteasome adaptor protein based on its structural composition and its rather small pore facilitating the entry of substrates for degradation^7,31,32^. PA200 is mainly composed of HEAT repeats, a repetitive array of amphiphilic alpha helices, which allow highly flexible and elastic conformational changes when interacting with different binding partners^33^. HEAT repeats facilitate protein-protein interactions and proteins harboring this structural motif are proposed to execute anchoring or adaptor functions^34^. Interestingly, the structurally related 200 kDa protein ECM29, which is also composed of multiple HEAT repeats and interacts with the 26S proteasome, was also proposed to function as an adaptor protein shuttling proteasomes to molecular motors and endosomes^35,36^. Adaptor proteins act as important temporal and spatial mediators of signaling cascades by transferring signals to specific protein complexes or subcellular compartments^37,38^. It is thus tempting to speculate that PA200 recruits proteasomes to distinct cellular compartments and thereby indirectly regulates proteasomal protein degradation. We have previously shown that the activity of the 26S proteasome is essential for myofibroblast differentiation^18^. As TGF-β1 promoted the formation of PA200-containing 26S proteasome complexes in phLF these complexes might be required for fine-tuning TGF-β1-induced myofibroblast differentiation. This may also involve chromatin regulation as PA200 has been shown to be recruited to chromatin upon DNA stress and to be involved in the degradation of acetylated histones^14,16^.

In conclusion, we here report an unexpected regulation of myofibroblast activation by PA200 and for the first time observe its regulation in disease. Using validated antibodies our study indicates the limited use of PA200^−/−^ mice and contributes to an increased understanding of PA200 regulation and function.

## Materials and Methods

All methods used in this study were carried out in accordance with the relevant guidelines and regulations.

### Human IPF and donor lung tissues

Human lung tissues from 13 IPF patients and 9 organ donors were used for mRNA/protein expression analyses including immunohistochemical studies and have previously been described^18^. Additionally, lung tissue samples of 4 donors (mean age ± SD: 46.5 ± 4.80 years; 3 females, 1 male) and from histologically normal areas of surgical lung specimens from 2 patients undergoing resective surgery for benign or malignant tumors (66.00 ± 8.48 years; 2 females) were used for isolation of normal human lung fibroblasts. All lung tissue samples were collected within the European IPF registry (eurIPFreg) and provided by the UGMLC Giessen Biobank (member of the DZL Platform Biobanking). The study protocol was approved by the Ethics Committee of the Justus-Liebig-University Giessen (No. 111/08 and 58/15), and informed consent was obtained in written form from each subject. All methods were carried out in accordance with the relevant guidelines and regulations.

### PA200^−/−^ mouse strain

Frozen sperm of PA200^−/−^ mice was provided by the laboratory of Barry Sleckman^29^. The PA200^−/−^ mouse strain was generated by *in vitro* fertilization of C57BL/6N mice and embryo transfer. Animals were kept at constant temperature and humidity with a 12 h light cycle and water and food *ad libitum*.

### Bleomycin-induced lung fibrosis

All animal studies conducted for this study were performed in accordance with international guidelines and were approved by the regional government of Upper Bavaria (animal approval file number 55.2-1-54-2532-114-2016). Lung fibrosis was induced in pathogen-free mice at the age of 10–12 weeks by instillation of a single dose of bleomycin (2 or 3 U/kg body weight depending on the batch) (Sigma Aldrich) to the lungs of age- and sex-matched mice as previously reported^18^.

### Oxalate-induced kidney fibrosis

Seven to eight week old female C57Bl6N mice were fed either a calcium-free control diet or oxalate-rich diet (prepared by mixing 50 µmol/g sodium oxalate in calcium-free control diet) for 21 days to induce fibrotic remodeling of the kidneys as previously reported^21^.

### Isolation and culture of primary human lung fibroblasts

Primary human lung fibroblasts (phLF) from non-diseased control lungs were isolated and cultured as described previously^39^. phLF between passage 3–5 were used for experiments. phLF were synchronized in starvation medium supplemented with 1% FBS 24 h prior to treatment with 5 ng/mL TGF-β1 (240-B-002, R&D Systems) for 24 h or 48 h. For analysis of proliferation, cells were harvested with trypsin, mixed with trypan blue (Sigma-Aldrich) and counted using a Neubauer counting chamber.

### Isolation and differentiation of primary human basal cells

Primary human bronchial epithelial cells were obtained from Lonza (Wokingham, UK) or isolated from airway tissue resected from patients undergoing lung surgery as described previously^22^. Isolated basal cells were *in vitro* differentiated for 28 days to a fully reconstituted epithelium, as described previously^22^.

### PA200 gene silencing

Knockdown of PA200 in primary human and mouse lung fibroblasts was performed by reverse transfection of *PSME4 (human)* siRNAs (*Silencer®* Select s23262 and s23263, Ambion, Life Technologies) and Psme4 (mouse) siRNAs (*Silencer®* Select s98098 and s98099), or control siRNAs (*Silencer®* Select Negative Control No. 1, 4390843, and Negative Control No. 2, 4390847, Ambion, Life Technologies) at a final concentration of 10 nM using Lipofectamine RNAiMAX (13778150, Life Technologies) as previously reported^18^.

### PA200 overexpression

phLF were transfected with a PA200 pcDNA3.1 expression vector by forward transfection using the Lipofectamine LTX reagent (Thermo Fisher Scientific). 24 h after seeding phLF, the transfection mix was prepared in OptiMEM according to the manufacturer’s protocol and added dropwise to cells. Medium was replaced with culture medium after 5 h and cells were harvested after 24 h hours. For TGF-β1 treatment, cell culture medium was replaced by 1% FBS starvation medium supplemented with 5 ng/mL TGF-β1 24 h after transfection and cells were harvested 24 h later.

### Native gel analysis

For generation of native protein extracts frozen tissues were homogenized twice for 30 s at 3000 rpm using a Mikro-Dismembrator S (Sartorius) prior to lysis under non-denaturing conditions. Lysis of tissue powder or cell pellets under native conditions in TSDG buffer and native gel analysis was performed as described before^40^.

### Western blot analysis

Cells or tissue homogenates were lysed in TSDG or RIPA buffer. SDS-PAGE and Western blot analysis were performed as described^40^. Membranes were incubated overnight at 4 °C with primary antibodies listed in Supplementary Table 1. Equal protein loading was monitored using an antibody directed against β-Actin coupled to HRP (A3854, 1:80 000, Sigma). HRP-linked anti-mouse IgG (7076S, 1:40 000, Cell Signaling) and anti-rabbit IgG secondary antibodies (7074S, 1:40 000, Cell Signaling) were used for detection. Blot membranes were developed with a chemiluminescent detection system (Merck Millipore), and emitted signals were detected using X-ray films. Densitometric analysis was performed in the linear range of exposure.

### Quantitative real-time PCR

RNA from cells or tissue powder was isolated using the Roti®-Quick-Kit (Carl Roth). RNA extracted from tissue powder was purified using the Peqlab-Gold Total RNA-Kit (12-6834-01, Peqlab). Reverse transcription and quantitative RT-PCR using the SYBR Green LC480 system (Roche) was conducted as previously described^41^. Primers are listed in Supplementary Table 2 and *RPL19* or *HPRT* served as housekeeping genes for relative gene expression analysis.

### Immunohistochemistry

Immunohistochemistry of paraffin-embedded IPF and donor lung tissues was performed as previously reported [18]. For staining of PA200 in paraffin-embedded murine testis tissues, sections were deparaffinized and permeabilized in methanol/hydrogen peroxide (80%/1.8% (v/v)) for 20 min. Heat-induced antigen retrieval was performed in citrate buffer pH 6 using a decloaking chamber (Biocare Medical) and unspecific binding sites were blocked with Rodent Block M (Biocare Medical) for 30 min. Slides were incubated with primary antibody diluted in Antibody Diluent (DAKO) for 1 h at RT. Afterwards, slides were incubated with MACH 2 Rabbit AP-Polymer (Biocare Medical) for 30 min at RT and Vulcan Fast Red AP substrate solution (Biocare Medical) for 10 min. Nuclei were stained with hematoxylin and slides were imaged using the MIRAX scanning system (Zeiss).

### Statistical analysis

Statistical analysis was performed with GraphPad Prism 5 and 7. Applied statistical tests are indicated in figure legends. P-values < 0.05 were considered statistically significant with *P < 0.05, **P < 0.01 and ***P < 0.001. Experiments with primary human lung fibroblasts were always performed in biological replicates with cells from different organ donors.

Other experimental procedures are described in the Supplementary Information.

## Supplementary information

Supplementary Information

## Data Availability

All data generated or analysed during this study are included in this published article (and its Supplementary Information files). Materials, data and associated protocols will be made available to readers without undue qualifications in material transfer agreements.
