# Supplementary material for: Proteasome activator PA200 regulates myofibroblast differentiation
Source: Sci Rep. 2019 Oct 23;9:15224. doi: 10.1038/s41598-019-51665-0 (PMC6811633; doi:10.1038/s41598-019-51665-0)
Supplement: Supplementary file 1 — Supplementary Information [file 41598_2019_51665_MOESM1_ESM.docx]

**Supplementary Information**

**Proteasome activator PA200 regulates myofibroblast differentiation**

Vanessa Welk^1^, Thomas Meul^1^, Christina Lukas^1^, Ilona E. Kammerl^1^, Shrikant R. Mulay^2^, Andrea C. Schamberger^1^, Nora Semren^1^, Isis E. Fernandez^1^, Hans-Joachim Anders^2^, Andreas Günther^3,4^, Jürgen Behr^1,5,6^, Oliver Eickelberg^1,7^, Martina Korfei^3^, and Silke Meiners^1*^

*Corresponding author: Silke Meiners, Max-Lebsche Platz 31, 81377 Munich, Germany, Phone: 00498931874673, Fax: 0049893187194673, e-mail: silke.meiners@helmholtz-muenchen.de

**Supplementary material and methods**

*Cell culture*

Human A549 cells were cultured in DMEM + GlutaMax (Thermo Fisher Scientific) supplemented with 10% (v/v) fetal bovine serum (FBS) (Biochrome) and 100 U/mL penicillin/streptomycin (Thermo Fisher Scientific).

*Proteasome activity assay*

Chymotrypsin- and caspase-like activities of the proteasome were measured with the Proteasome-Glo^TM^ Assay kit (Promega) using the luminescent substrates Suc-LLVY-aminoluciferin specific for chymotrypsin-like and Z-nLPnLD-aminoluciferin specific for caspase-like activities. Proteasome activity of 2 µg of protein/well from a native protein extract was analyzed in three technical replicates according to the manufacturer’s protocol. Luminescence was measured every 2 min for 30 min using a TriStar LB 941 plate reader (Berthold Technologies) and values reaching the plateau were used for quantification of proteasome activities.

*Masson-Trichrome staining*

Masson-Trichrome staining was performed with the Trichrome Stain (Masson) kit (HT15, Sigma-Aldrich). Murine kidney sections were deparaffinized and incubated in Bouin’s solution (Sigma-Aldrich) at RT overnight. The staining was performed according to the manufacturer’s protocol. Slides were rehydrated, dried, mounted with Entellan mounting medium (Merck Millipore) and imaged using the Mirax scanning system (Zeiss).

**Supplementary Tables**

*Supplementary Table 1: Antibodies used in the study*

| **Antigen** | **Product number** | **Host** | **Type** | **Appli-cation** | **Dilution** | **Provider** |
| --- | --- | --- | --- | --- | --- | --- |
| COL1A1 | 600401103 | Rabbit | Polyclonal | WB | 1:5000 | Rockland Immunochemicals |
| Cyclin D1 | 2978 | Rabbit | Monoclonal | WB | 1:1000 | Cell Signaling |
| Fibronectin | sc-9068 | Rabbit | Polyclonal | WB | 1:1000 | Santa Cruz |
| GAPDH  (HRP-linked) | 14C10 | Rabbit | Monoclonal | WB | 1:80 000 | Cell Signaling |
| KRT5 | ab75869 | Rabbit | Polyclonal | IHC | 1:150 | Abcam |
| Normal rabbit IgG | 2729 | Rabbit | Polyclonal | IHC | IgG ctrl | Cell Signaling |
| PA200 (antibody #1) | PA1-1961 | Rabbit | Polyclonal | WB | 1:1000  1:600 | Thermo Fisher Scientific |
| PA200 (antibody #2; for human) | NBP1-22236 | Rabbit | Polyclonal | WB | 1:2500 | Novus Biologicals |
| PA200 (antibody #3; for mouse) | NBP2-32575 | Rabbit | Polyclonal | WB | 1:500 | Novus Biologicals |
| PA200 (antibody #4; mouse and human) | sc-135512 | Rabbit | Polyclonal | IHC | 1:50 | Santa Cruz |
| PCNA | 18-0110 | Mouse | Monoclonal | WB | 1:2000 | Thermo Fisher Scientific |
| RPT5 | A303-538A | Rabbit | Polyclonal | WB | 1:5000 | Bethyl Laboratories |
| α1-7 (MCP231) | ab22674 | Mouse | Monoclonal | WB | 1:1000 | Abcam |
| αSMA | A5228 | Mouse | Monoclonal | WB | 1:1000 | Sigma Aldrich |
| αSMA | ab5694 | Rabbit | Polyclonal | IHC | 1:200 | Abcam |
| β5 | ab90867 | Rabbit | Polyclonal | WB | 1:1000 | Abcam |
| β-Actin (HRP-linked) | A3854 | Mouse | Monoclonal | WB | 1:80000 | Sigma Aldrich |

*Supplementary Table 3: Primers used for RT-qPCR*

| **Gene** | **Species** |  | **Sequence 5’-3’** |
| --- | --- | --- | --- |
| *ACTA2* | human | FW | CGAGATCTCACTGACTACCTCATGA |
|  |  | REV | AGAGCTACATAACACAGTTTCTCCTTGA |
| *CCND1* | human | FW | CGTGGCCTCTAAGATGAAGG |
|  |  | REV | CTGGCATTTTGGAGAGGAAG |
| *COL1A1* | human | FW | CAAGAGGAAGGCCAAGTCGAG |
|  |  | REV | TTGTCGCAGACGCAGATCC |
| *HPRT* | human | FW | TGAAGGAGATGGGAGGCCA |
|  |  | REV | AATCCAGCAGGTCAGCAAAGAA |
| *PSME4* | human | FW | CCAACAGGAAAAGAATGCCGA |
|  |  | REV | CCAGGGCAGGTTTCTTTGCT |
| *RPL19* | human | FW | TGTACCTGAAGGTGAAGGGG |
|  |  | REV | GCGTGCTTCCTTGGTCTTAG |
| *TGFB1* | human | FW | CGACTCGCCAGAGTGGTTAT |
|  |  | REV | TAGTGAACCCGTTGATGTCCA |
| *Acta2* | mouse | FW | GCTGGTGATGATGCTCCCA |
|  |  | REV | GCCCATTCCAACCATTACTCC |
| *Col1a1* | mouse | FW | CCAAGAAGACATCCCTGAAGTCA |
|  |  | REV | TGCACGTCATCGCACACA |
| *Fn* | mouse | FW | GTGTAGCACAACTTCCAATTACGAA |
|  |  | REV | GGAATTTCCGCCTCGAGTCT |
| *Hprt* | mouse | FW | AATCCAGCAGGTCAGCAAAGAA |
|  |  | REV | TGAAGGAGATGGGAGGCCA |
| *Psme4* | mouse | FW | CATCCTTCAAATAATGGGCG |
|  |  | REV | AAGCTTATGGCTTTCAGGCA |
| *Rpl19* | mouse | FW | CGGGAATCCAAGAAGATTGA |
|  |  | REV | TTCAGCTTGTGGATGTGCTC |
| *Tnc* | mouse | FW | GCTTCACTGGCAAAGACTGCAA |
|  |  | REV | CGTAAAGCCCTCATGGCAGATA |

**
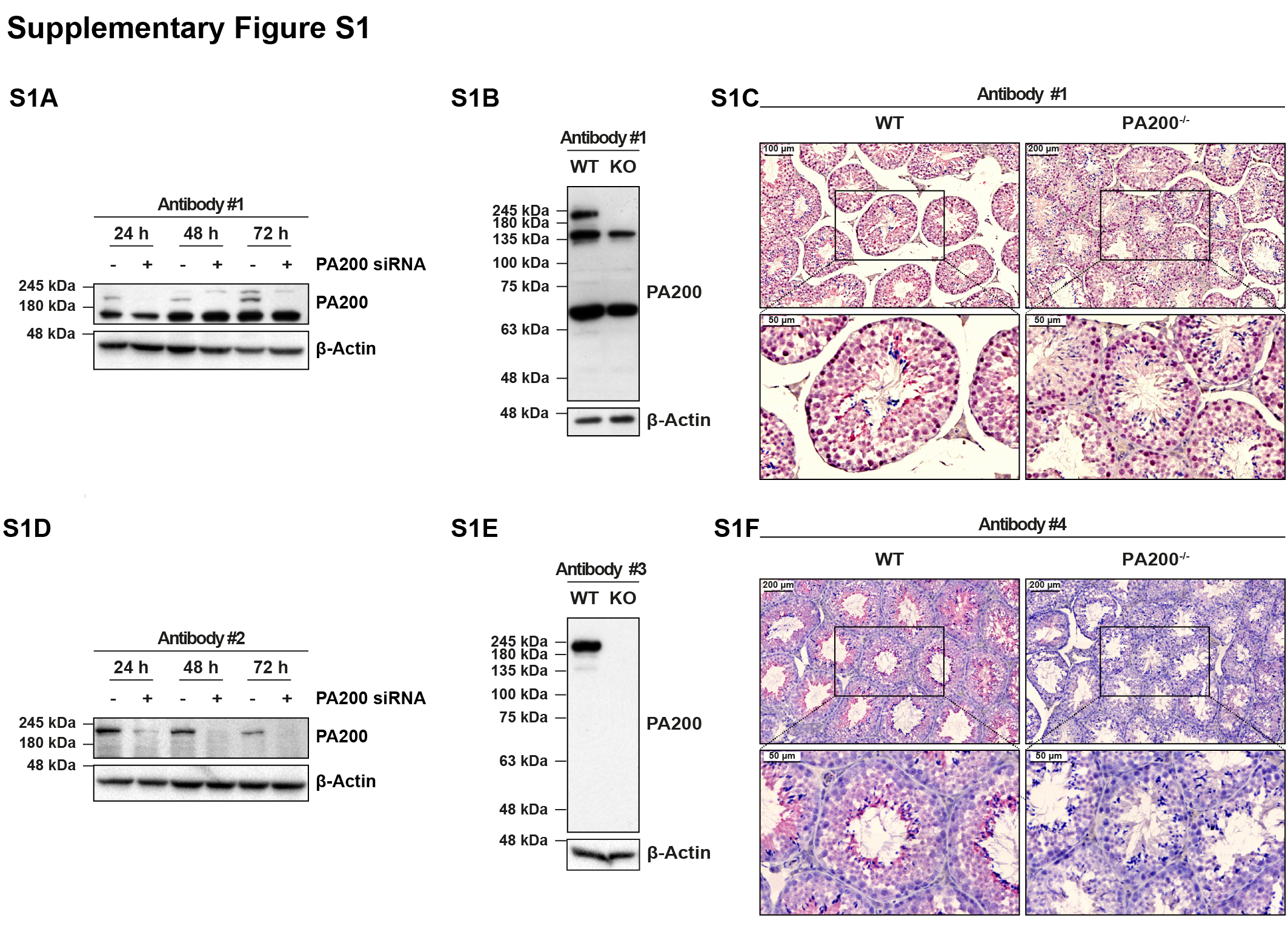
**

*Supplementary Figure S1*

S1A) A time course of transient PA200 silencing in human A549 alveolar epithelial cells was analyzed for specific recognition of PA200 by Western blotting using antibody #1 (PA1-1961, Thermo Fisher Scientific). S1B) Total testis homogenates of wildtype (WT) and PA200^-/-^ (KO) mice were analyzed for specific detection of PA200 by Western blotting using antibody #1. S1C) Immunohistochemistry (IHC) staining of paraffin-embedded testis sections from wildtype (WT) and PA200^-/-^ (KO) mice with antibody #1. S1D) Samples used in S1A) were analyzed with the specific PA200 targeting antibody #2 (NBP1-22236, Novus Biologicals). S1E) Samples used in S1B) were analyzed with the specific PA200 targeting antibody #3 (NBP2-32575, Novus Biologicals). S1F) Tissue sections from wildtype (WT) and PA200^-/-^ (KO) mice were stained with antibody #4 (sc-135512, Santa Cruz) by IHC. Figures show representative results for experiments performed with *n* = 3.

*
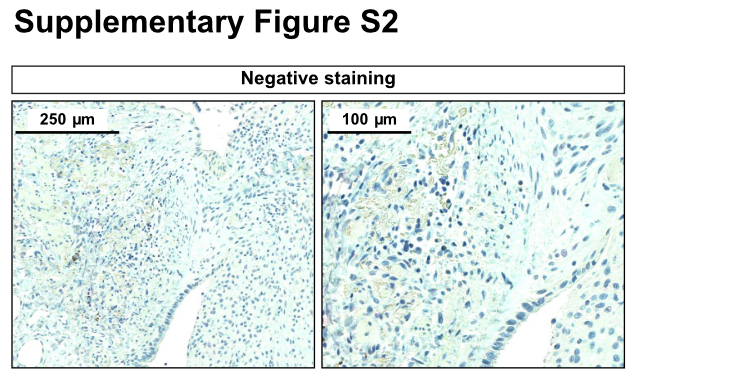
*

*Supplementary Figure S2*

Negative staining of IPF lung tissue sections without primary antibody was performed together with immunohistochemistry analysis shown in Figure 1B in order to control for unspecific staining

*
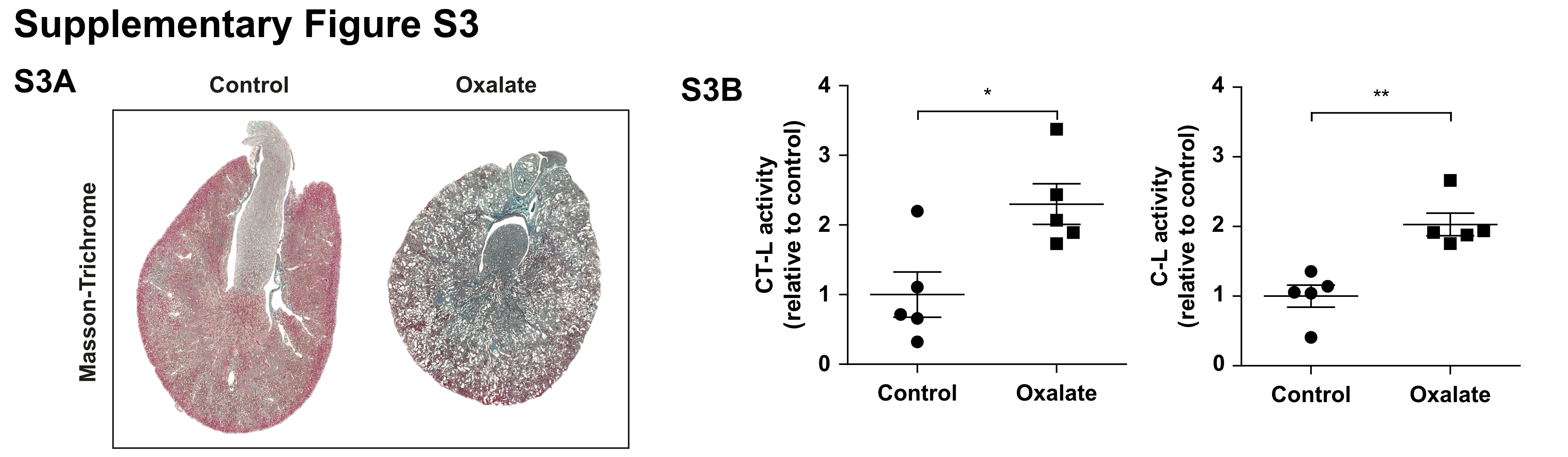
*

*Supplementary Figure S3*

S3A) Representative Masson-Trichrome staining of control- and oxalate-treated mice (50 µmol/g sodium oxalate in a standard diet for 21 days) showing collagen deposition (blue) in fibrotic kidney. S3BC) Chymotrypsin-like (CT-L) and caspase-like (C‑L) activities of the proteasome were analyzed in the same native total kidney extracts as used for native gel electrophoresis in Figure 2D. Diagrams show activities normalized to the mean of controls (Mann‑Whitney test, *n =* 5 per group).

*
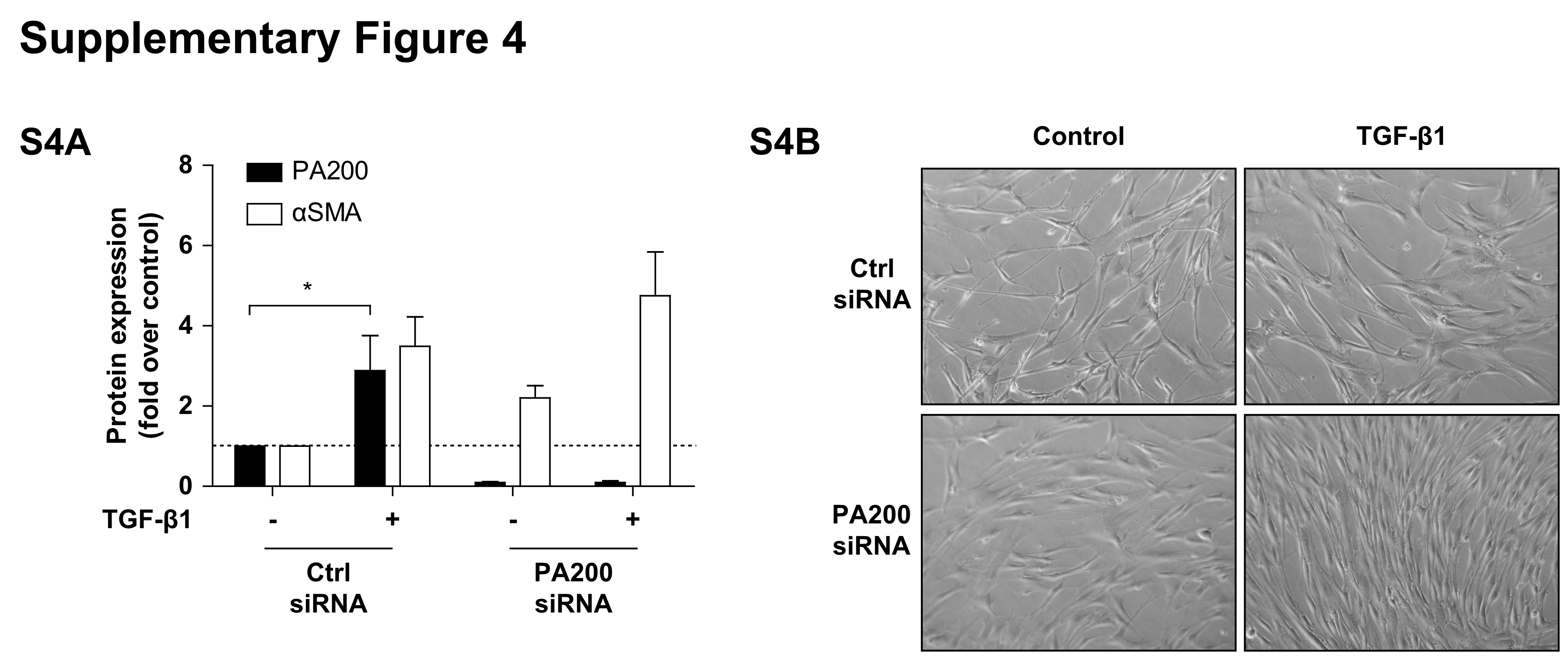
*

*Supplementary Figure S4*

S4A) Densitometric analysis of Western blots as shown in Figure 6A (Bonferroni’s multiple comparison test, phLF from *n* = 4 different organ donors). S4B) Representative images taken of phLF as analyzed in Figure 6B at time point of harvest.


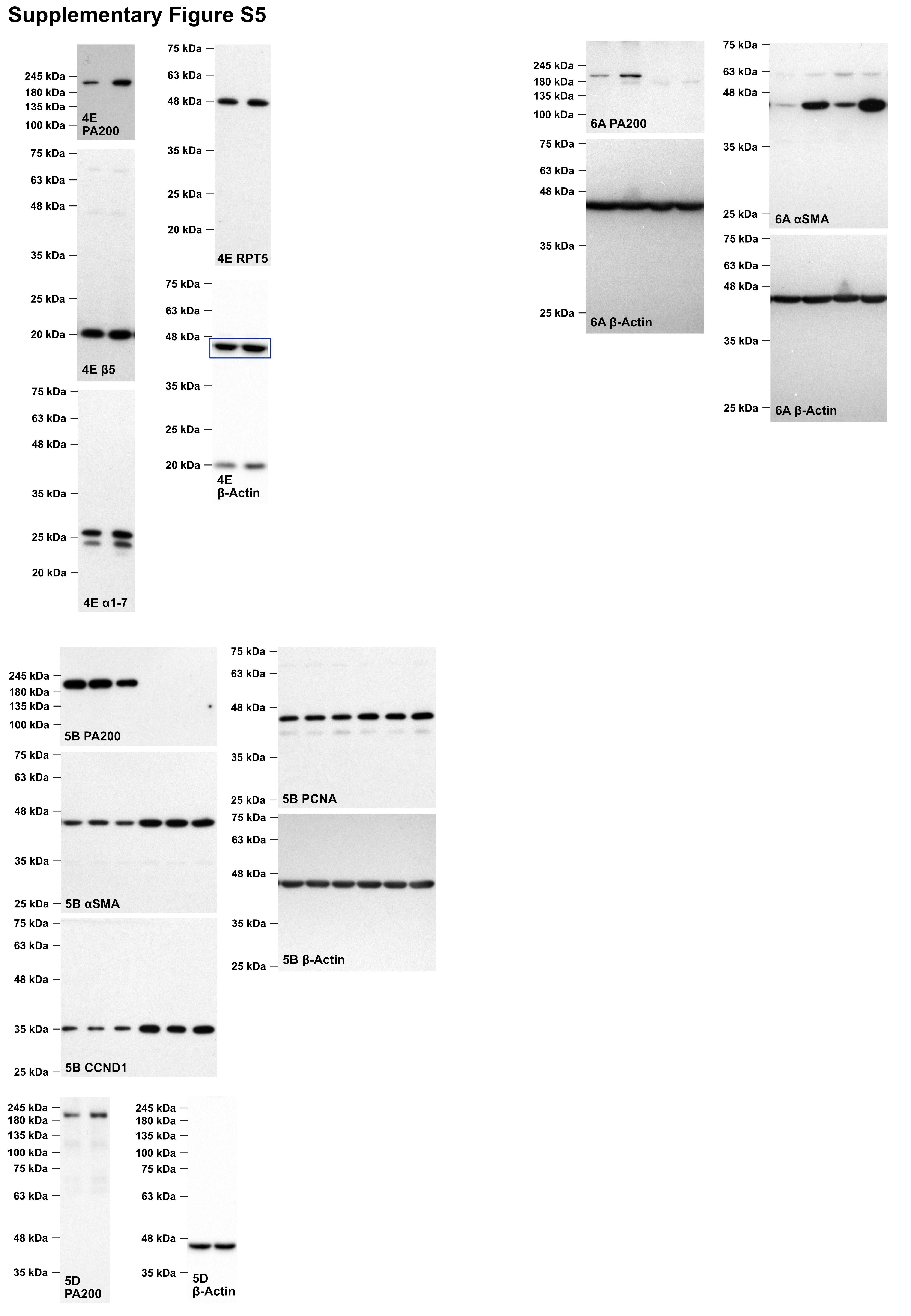

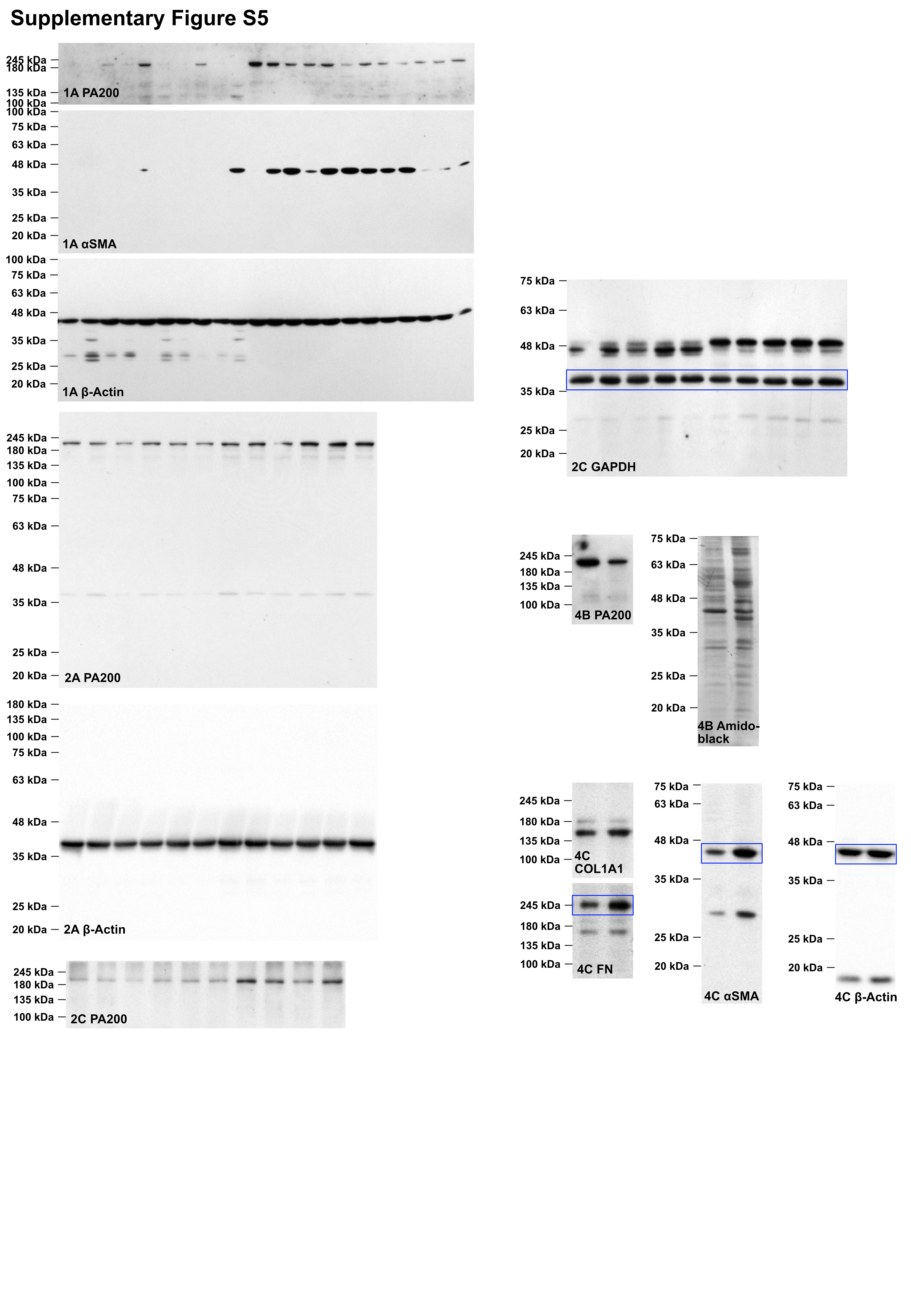


*Supplementary Figure S5*

*Uncropped immunoblots for the respective figures with indicated protein bands. Bands used for quantification are marked with blue boxes in case several bands are visible.*

**Supplementary Figure S6**

*Supplementary Figure S6*

mRNA expression analysis of PA200 (Psme4), TGF-β1 (Tgfb1) and αSMA (Acta2) in pmLF after 24, 48 or 72 h of PA200 silencing. Hprt served as housekeeping gene and expression was normalized to time-matching controls (one-sample t-test, pmLF from four different wildtype mice).
